# Inflammation and poverty as individual and combined predictors of 15-year mortality risk in middle aged and older adults in the US

**DOI:** 10.3389/fmed.2023.1261083

**Published:** 2024-01-16

**Authors:** Arch G. Mainous, Frank A. Orlando, Lu Yin, Pooja Sharma, Velyn Wu, Aaron Saguil

**Affiliations:** ^1^Department of Community Health and Family Medicine, University of Florida, Gainesville, FL, United States; ^2^Department of Health Services Research Management, and Policy, University of Florida, Gainesville, FL, United States

**Keywords:** National Health and Nutrition Examination Survey, mortality, cohort, poverty, inflammation

## Abstract

**Background:**

Chronic systemic inflammation and poverty are both linked to an increased mortality risk. The goal of this study was to determine if there is a synergistic effect of the presence of inflammation and poverty on the 15-year risk of all-cause, heart disease and cancer mortality among US adults.

**Methods:**

We analyzed the nationally representative National Health and Nutrition Examination Survey (NHANES) 1999 to 2002 with linked records to the National Death Index through the date December 31, 2019. Among adults aged 40 and older, 15-year mortality risk associated with inflammation, C-reactive protein (CRP), and poverty was assessed in Cox regressions. All-cause, heart disease and cancer mortality were the outcomes.

**Results:**

Individuals with elevated CRP at 1.0 mg/dL and poverty were at greater risk of 15-year adjusted, all-cause mortality (HR = 2.45; 95% CI 1.64, 3.67) than individuals with low CRP and were above poverty. For individuals with just one at risk characteristic, low inflammation/poverty (HR = 1.58; 95% CI 1.30, 1.93), inflammation/above poverty (HR = 1.59; 95% CI 1.31, 1.93) the mortality risk was essentially the same and substantially lower than the risk for adults with both. Individuals with both elevated inflammation and living in poverty experience a 15-year heart disease mortality risk elevated by 127% and 15-year cancer mortality elevated by 196%.

**Discussion:**

This study extends the past research showing an increased mortality risk for poverty and systemic inflammation to indicate that there is a potential synergistic effect for increased mortality risk when an adult has both increased inflammation and is living in poverty.

## Introduction

Systemic inflammation is associated with the development and progression of many chronic conditions, cardiovascular (CVD), metabolic, renal and oncologic diseases, as well as morbidity and mortality (1–3). Evidence has accumulated indicating the significant relevance of low-grade inflammatory processes to cardiovascular disease, cancer and vascular risk factors (1, 4). Further, high sensitivity C-reactive protein (hs-CRP) is a strong independent predictor of future cardiovascular events (3, 5). Cohort studies have shown that elevated CRP is associated with mortality and cardiovascular disease (CVD) events for patients with various CVD locations like coronary artery disease, cerebrovascular disease, peripheral artery disease, and abdominal aortic aneurysm (6).

Risk factors such as age, diet, lifestyle, and environmental pollutants impact the biochemical and genetic pathways that lead to states of chronic inflammation (1, 4, 7–9). In patients with known cardiovascular disease (CVD), elevated CRP is associated with an increased risk for future CVD events and mortality (6). Elevated CRP is associated with elevated all-cause mortality risk (10–13). One meta-analysis has linked elevated CRP to both all-cause and CVD mortality risk (14).

An estimated 37.9 million Americans (11.6%) were living in poverty in 2021 (15). Poverty negatively affects the health of individuals (16). Poverty correlates with lower life expectancy and premature mortality risk (17–19). Poverty is also linked to increased inflammation (20–21). However, because poverty and inflammation are correlated and both associated with mortality risk but at the same time independent of each other, it is unclear if they act synergistically for mortality and in particular, heart disease and cancer mortality.

This cohort study will provide US population estimates of 15-year mortality risk for the individual and combined presence of poverty and systemic inflammation among middle age and older adults with baseline assessments of poverty and systemic inflammation.

## Methods

This study is an analysis of the publicly available, deidentified National Center for Health Statistics NHANES data linked to the National Death Index by the National Center for Health Statistics. We analyzed the National Health and Nutrition Examination Survey (NHANES) 1999 to 2002 with linked records to the National Death Index (NDI) through the date December 31, 2019. The NHANES uses a stratified multistage probability sample design to be representative of the United States (US) population. Participation in NHANES includes providing information through surveys, physical and physiologic examinations, and laboratory assays. The 1999 to 2002 baseline NHANES sample included 3,478 unweighted participants aged 40 and older. We limited the baseline cohort to individuals assessed in the four-year NHANES data collection period of 1999–2002. We had that group as our cohort so that included individuals would be available to be followed for 15-year mortality risk by the end of the available NDI data. The NHANES is an ongoing survey and later years are available but the later years would not allow for linking to the NDI and still allow a 15 year follow-up. As a population-based cohort with a complex survey design and appropriate weighting the design provides a population estimate representative of the noninstitutionalized US population.

### Study cohort definition

The individuals included in this study cohort were aged 40 and older at baseline. Patients were included if they participated in the NHANES 1999–2002 and had the key variables of inflammation, poverty and associated demographics. There was no blinding in this retrospective cohort and the linkage of the data from the NHANES to the NDI was provided by the National Center for Health Statistics and released as a deidentified, public use database. By using middle aged and older adults at baseline it improves the ability to focus on downstream mortality over the next 15 years.

### Inflammation

Inflammation was defined at baseline by means of CRP levels. The NHANES reported CRP levels for all of the participants in our study. We categorized elevated inflammation in two different ways. First, elevated CRP was defined as >0.3 mg/dL, as recommended by the Centers for Disease Control and Prevention and the American Heart Association (5). This level was based on evidence of chronic systemic inflammation and CVD risk. Second, in an additional analysis we defined elevated CRP as >1.0 mg/dL which is consistent with systemic inflammation (22).

### Poverty

Poverty was defined according to the poverty index ratio which is a standard measure of total family income divided by the poverty threshold. The poverty threshold accounts for the size of the family and the number of related children in the household under 18. Poverty at baseline simply defines whether the individual was living below the poverty line as a baseline exposure characteristic in 1999 to 2002. We categorized individuals in the sample into two groups: (a) Persons without poverty at baseline (“above poverty”) had a poverty income ratio above 1 indicating that the person was not in poverty at baseline and, (b) Persons with poverty at baseline (“poverty”) had a poverty income ratio at or below 1 indicating that the person was in poverty at baseline.

### Mortality

The National Center for Health Statistics (NCHS) has linked data collected from the NHANES with death certificate records from the National Death Index (NDI). The mortality status for each participant was censored at 15 years to create consistency among follow-up lengths between members of the different NHANES cohorts. This study used the public use linked mortality files for the nine cause-specific death categories produced by the NCHS [Public-use Linked Mortality File Readme (cdc.gov)]. The NCHS recoded 113 underlying causes of death into several categories. We examined all-cause mortality, heart disease mortality and cancer mortality.

### Analysis

We classified the population into 4 groups based on inflammation and poverty (above poverty/low inflammation; above poverty/elevated inflammation; poverty/low inflammation; and poverty/elevated inflammation). We used sampling weights to calculate prevalence estimates for the civilian noninstitutionalized US population. All analyses were conducted using the survey package in R 4.3.3 to account for the complex NHANES sampling design and make population estimates. Thus, the analysis represented a population of approximately 95 million people.

Using the population estimates, we graphically show the cumulative mortality as the unadjusted relationship by the 4 inflammation/poverty groups. We performed Cox proportional hazards analysis with mortality time for each group, controlling for age, sex, and race/ethnicity. We defined elevated inflammation in one analysis as CRP >0.3 mg/dL and in a second analysis elevated inflammation was defined as >1.0 mg/dL. We used as the outcomes 15-year risk of all-cause mortality, heart disease and cancer mortality.

## Results

**Figure 1:**
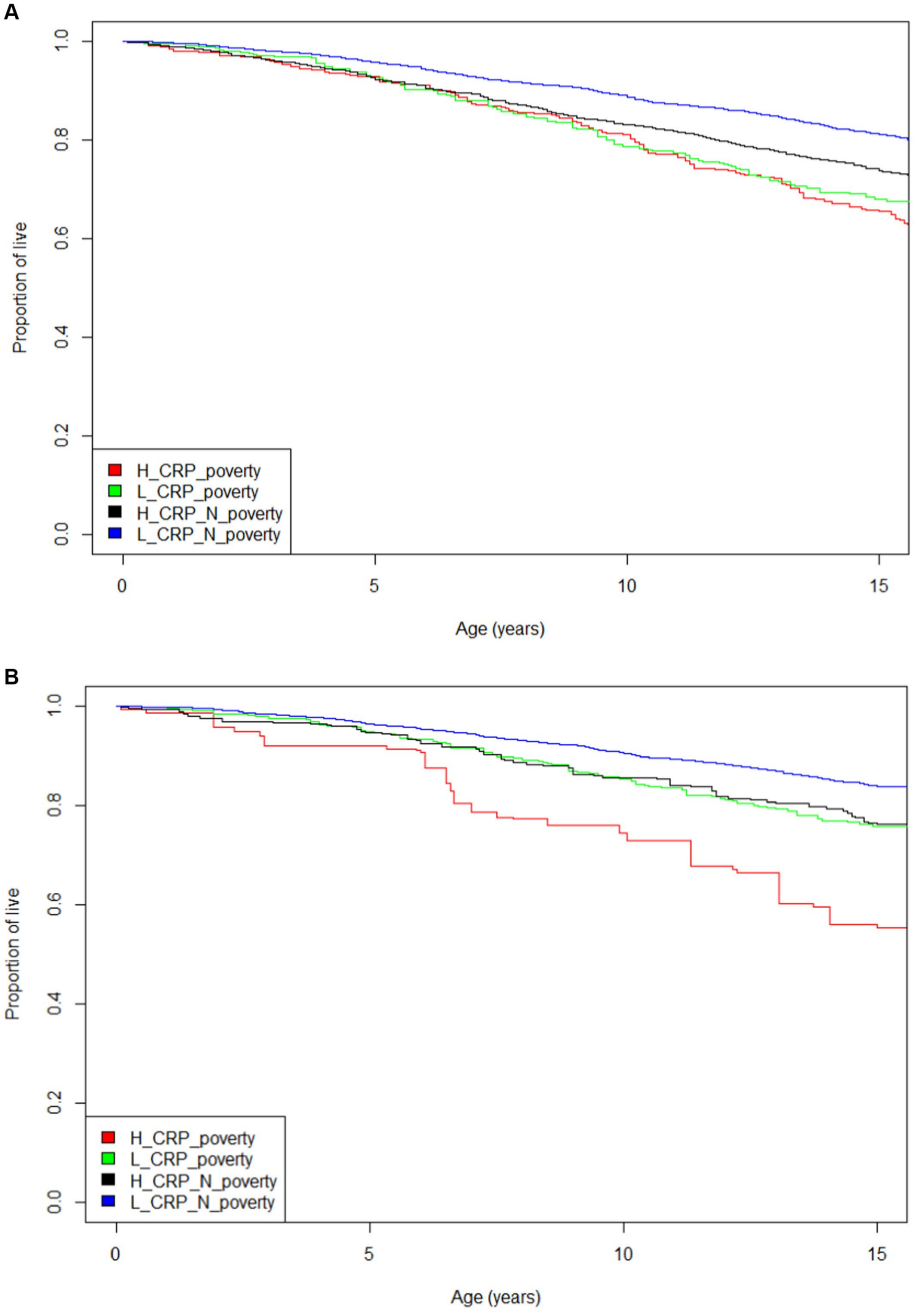
**(A)** Mortality Kaplan–Meier curve for four inflammation and poverty groups and 15 year mortality with CRP = 0.3 mg/dL (log rank test *p* < 0.001). **(B)** Mortality Kaplan–Meier curve for four inflammation and poverty groups and 15 year mortality with CRP =1.0 mg/dL (log rank test *p* < 0.001).

The characteristics of the sample are shown in Table 1. The individuals who live in poverty account for 11.4% of the population. Figure 1 presents the Kaplan–Meier curves for all four groups using two different CRP cutpoints. Figure 1A has a CRP cut off of 0.3 mg/dL to define high inflammation. Figure 1B shows a CRP cut off of 1.0 mg/dL to define high inflammation. These displays of the relationship between inflammation/poverty and mortality over 15 years are unadjusted for variables like age and race/ethnicity, but they convey the general mortality risk. In particular, when using a CRP cut off of 1.0 mg/dL, the above poverty/low inflammation group has lower mortality than the two intermediate groups (high inflammation/above poverty and low inflammation/high poverty) while the poverty/high inflammation group has the highest mortality over 15 years.

**Table 1:** Population estimates for demographic characteristics of all four groups among adults aged ≥ 40 years with CRP at 0.3 mg/dL, 1999–2002 (Unweighted *N* = 4,849; Weighted *N* = 94,821,664).

Characteristics	Low inflammation/above poverty	High inflammation/above poverty	Low inflammation/poverty	High inflammation/poverty	Value of *p*
Unweighted sample size	2,287	1766	389	407	
Weighted population size	50,031,662	33,893,028	5,449,080	5,447,894	
Sex (%)					
Male	56	39	47	35	
Female	44	61	53	65	
Age (%)					<0.001
40–49 years	44	32	42	36	
50–59 years	27	29	20	28	
60–69 years	16	21	17	15	
70–79 years	11	13	16	18	
80 years & above	3.4	4.2	4.5	3.4	
Race/ethnicity (%)					<0.001
Non-Hispanic White	82	79	55	50	
Non-Hispanic Black	6.5	9.9	13	19	
Mexican American	8.2	9.0	24.2	21.6	
Other Race	3.3	1.9	6.8	10.0	

The adjusted Cox proportional hazard analysis for mortality risk featured in Table 2 confirmed the trends seen in the unadjusted Kaplan–Meier curves. The results in the analysis with inflammation defined as CRP 0.3 mg/dL suggests that individuals with high CRP levels are at basically equal increased mortality risk whether they are living in poverty or are above the poverty level. However, the analysis which defines inflammation as CRP at 1.0 mg/dL shows that there is a synergistic effect on mortality risk when a person has both elevated inflammation and is living in poverty.

**Table 2:** Cox regression model for all cause-mortality risk among the 4 groups at CRP 0.3 and 1.0 mg/dL.

All-cause mortality	Hazard ratio at CRP 0.3 mg/dL		Hazard ratio at CRP 1.0 mg/dL	
	Unadjusted (95%CI)	Adjusted (95%CI)**	Unadjusted (95%CI)	Adjusted (95%CI)**
Low inflammation/above poverty line*	--	--	--	--
Low inflammation/poverty	1.47 (1.28–1.68)	1.35 (1.17–1.55)	1.55 (1.30–1.84)	1.58 (1.30–1.93)
Hi inflammation/above poverty	1.86 (1.33–2.60)	1.85 (1.53–2.24)	1.55 (1.22–1.98)	1.59 (1.31–1.93)
Hi inflammation/poverty	2.00 (1.60–2.50)	1.91 (1.43–2.54)	2.53 (1.68–3.81)	2.45 (1.64–3.67)

*Reference category; **Adjusted HR for age, sex, race/ethnicity.

In addition to the all-cause mortality analyses, we also conducted two another analyses for death from heart disease and cancer, respectively. Table 3 shows that when inflammation is defined as CRP 1.0 mg/dL, individuals with both elevated inflammation and living in poverty experience a 15-year heart disease mortality risk elevated by 127% and 15-year cancer mortality elevated by 196%.

**Table 3:** Cox regression model for heart disease and cancer mortality among the 4 groups at CRP 0.3 and 1.0 mg/dL.

	Hazard ratio at CRP 0.3 mg/dL		Hazard ratio at CRP 1.0 mg/dL	
	Unadjusted (95%CI)	Adjusted (95%CI)**	Unadjusted (95%CI)	Adjusted (95%CI)**
Heart disease				
Low inflammation/above poverty line*	--	--	--	--
Low inflammation/poverty	1.38 (1.06–1.78)	1.27 (0.94–1.71)	1.51 (1.08–2.11)	1.57 (1.10–2.23)
High inflammation/above poverty	1.67 (1.06–2.61)	1.82 (1.15–2.88)	1.54 (1.17–2.04)	1.71 (1.26–2.31)
High inflammation/poverty	1.95 (1.33–2.85)	1.97 (1.25–3.10)	2.20 (1.27–3.82)	2.27 (1.23–4.19)
Cancer				
Low inflammation/above poverty line*	--	--	--	---
Low inflammation/poverty	1.28 (0.91–1.81)	1.25 (0.87–1.81)	1.36 (0.94–1.97)	1.47 (1.00–2.16)
Hi inflammation/above poverty	1.60 (0.97–2.65)	1.78 (1.14–2.78)	1.13 (0.75–1.69)	1.29 (0.87–1.92)
Hi inflammation/poverty	1.37 (0.75–2.53)	1.53 (0.79–2.98)	2.64 (1.40–5.00)	2.96 (1.56–5.59)

*Reference category; **Adjusted HR for age, sex, race/ethnicity.

## Discussion

The results of this study reinforce the findings of previous research that both elevated systemic inflammation and poverty are risk factors for mortality. This study extends the past research to indicate that there is a potential synergistic effect for increased mortality risk when an adult has both increased inflammation and is living in poverty. This effect is specifically observed when inflammation is defined as CRP >1.0 mg/dL. However, inflammation is considered a modifiable risk but is not usually measured clinically except for certain autoimmune and infectious diseases. Therefore, screening high-risk populations for elevated hs-CRP and early initiation of anti-inflammatory treatment, potentially diet or even medications would contribute to the reduction of the risk of future disease and as is shown here, mortality.

This study is clinically relevant because both inflammation and poverty are modifiable risk factors. It emphasizes that focusing only on one of the variables, poverty or inflammation, will still not reduce the mortality risk to that of individuals living above the poverty level with no systemic inflammation. Inflammation and corresponding mortality risk could potentially be reduced by anti-inflammatory diets or potentially anti-inflammatory medications (23–26).

Impoverished social conditions and the underlying impetuses forming them are the basis for preventable disparities in various health outcomes (27). The diet among people in poverty as well as their stress levels contribute to a higher risk of systemic inflammation. It may be useful to target individuals living in poverty for screening for systemic inflammation. It is therefore imperative to also have a better clinical understanding of poverty’s relationship with chronic disease morbidity and mortality to guide future screening and outcome studies on social determinants of health.

In June 20, 2023, the US Food and Drug Administration approved colchicine as the first anti-inflammatory, atheroprotective cardiovascular treatment (28). Specifically, patients with systemic inflammation, as measured by hs-CRP, now have an FDA-approved treatment option demonstrated to reduce the risk of cardiovascular disease by targeting inflammatory pathways.

This study has several strengths and limitations. In terms of strengths, this is a population-based cohort that allows us to make estimates for the non-institutionalized adult population of the US. Second, the inflammation measures, CRP, are standard measures and were collected in a standardized way for everyone. They were not based on a patient being symptomatic, which would likely be the case in an analysis from an electronic health record.

There are some limitations to the study. First, as with any cohort study, there is a general assumption in observational studies that the baseline exposure variable (e.g., inflammation, poverty) has a certain degree of constancy or has had such a physiological insult to the person that it carries over to the downstream mortality risk. Several economic crises have occurred with a relevant impact on the United States population. The key variables in the study may have been affected by the so-called “cohort effect.” The findings may have been affected by that and may not be reliably representative of the current US population. In this case, since systemic inflammation is not universally recommended for screening at either CRP 0.03 mg/dL or CRP 1.0 mg/dL, it is unlikely that there would be any interventions to directly lower that variable. Similarly, unfortunately, many people tend to remain in poverty. Second, the cohort was assessed for 15-year mortality risk. It is possible that the risk may have increased if the follow-up period had been longer. However, 15-years among middle aged and older adults would generally capture premature mortality risk.

In conclusion, inflammation and poverty are well known risk factors for mortality, but when both exist simultaneously and CRP is >1.0 mg/dL, they have the potential to increase mortality more than one would expect from an additive effect. This is particularly concerning in socially disadvantaged patients who are already a medically vulnerable population. Moreover, elevated inflammation is not typically known in asymptomatic populations. Perhaps targeted screening for elevated CRP in vulnerable populations might be particularly useful. Even though both inflammation and poverty are modifiable risk factors, in clinical practice, chronic diseases associated with inflammation like cardiovascular disease are more likely to be prevented by healthy lifestyle than be reversed.

## Data availability statement

Publicly available datasets were analyzed in this study. This data can be found here: https://wwwn.cdc.gov/nchs/nhanes/continuousnhanes/default.aspx?BeginYear=1999.

## Author contributions

AM: Conceptualization, Project administration, Writing – original draft. FO: Conceptualization, Investigation, Writing – review & editing. LY: Formal analysis, Writing – review & editing. PS: Formal analysis, Writing – review & editing. VW: Conceptualization, Writing – review & editing. AS: Conceptualization, Writing – review & editing.
